# Doxorubicin resistance in breast cancer cells is mediated by extracellular matrix proteins

**DOI:** 10.1186/s12885-017-3953-6

**Published:** 2018-01-06

**Authors:** Carrie J. Lovitt, Todd B. Shelper, Vicky M. Avery

**Affiliations:** Discovery Biology, Griffith Institute for Drug Discovery, Griffith University, Building N27, Brisbane Innovation Park, Nathan, QLD 4111 Australia

**Keywords:** Doxorubicin, Extracellular matrix, Three-dimensional cell culture, Drug resistance

## Abstract

**Background:**

Cancer cell resistance to therapeutics can result from acquired or de novo-mediated factors. Here, we have utilised advanced breast cancer cell culture models to elucidate de novo doxorubicin resistance mechanisms.

**Methods:**

The response of breast cancer cell lines (MCF-7 and MDA-MB-231) to doxorubicin was examined in an in vitro three-dimensional (3D) cell culture model. Cells were cultured with Matrigel™ enabling cellular arrangements into a 3D architecture in conjunction with cell-to-extracellular matrix (ECM) contact.

**Results:**

Breast cancer cells cultured in a 3D ECM-based model demonstrated altered sensitivity to doxorubicin, when compared to those grown in corresponding two-dimensional (2D) monolayer culture conditions. Investigations into the factors triggering the observed doxorubicin resistance revealed that cell-to-ECM interactions played a pivotal role. This finding correlated with the up-regulation of pro-survival proteins in 3D ECM-containing cell culture conditions following exposure to doxorubicin. Inhibition of integrin signalling in combination with doxorubicin significantly reduced breast cancer cell viability. Furthermore, breast cancer cells grown in a 3D ECM-based model demonstrated a significantly reduced proliferation rate in comparison to cells cultured in 2D conditions.

**Conclusion:**

Collectively, these novel findings reveal resistance mechanisms which may contribute to reduced doxorubicin sensitivity.

## Background

Breast cancer has the highest incidence and mortality rate of all cancers in the female population [1]. Therapeutic options for the treatment of breast cancer are dependent on the specific biological characteristics of the tumour. If the tumour is low grade, node-negative and estrogen-receptor positive, hormone therapy may be recommended, however, if the tumour is of high grade and/or node-positive, chemotherapy is generally administered prior to targeted therapies depending on the hormonal/ErBb2 status of the tumour [2]. Anthracyclines, such as doxorubicin and epirubicin; taxanes, including paclitaxel and docetaxel, along with fluorouracil and cyclophosphamide are the current therapeutics utilised for combination adjuvant breast cancer treatment [3]. However, disease progression will occur in an estimated 20–30% of patients with early-stage disease following adjuvant therapy [4].

The principal actions of anthracyclines are DNA intercalation, inhibition of topoisomerase II and the formation of free radicals [5]. Resistance mechanisms specific to topoisomerase II inhibitors have been identified and include enhanced levels of efflux and alterations to the expression of the topoisomerase II [6]. Resistance to therapeutics can be caused by numerous factors, associated with either acquired or de novo mechanisms. Acquired mechanisms of resistance progress in response to exposure to the therapeutics, whereas de novo resistance relates specifically to the characteristics of a tumour that exist prior to the application of anti-cancer agents [7]. De novo resistance can be mediated by environmental influences, such as tumour cell attachment to elements of the stroma, including the extracellular matrix (ECM) [8, 9].

Traditionally, investigations into the activity and resistance mechanisms of new drug candidates have been conducted utilising two-dimensional (2D) cell culture conditions. However, these models do not incorporate the elements of a tumour, such as cell-to-extracellular matrix (ECM) interactions and a three-dimensional (3D) arrangement of cells, which are advantageous for evaluating the activity of anti-tumour therapeutics in vitro [10]. In a previous study, chemotherapeutic drugs were demonstrated to be less effective on specific cell lines cultured in the advanced culture conditions [11]. In addition, ECM-based 3D cell culture models have been utilised for elucidation of breast cancer cell resistance mechanisms following drug exposure [12, 13]. Utilising a 3D ECM-based model, we investigated doxorubicin resistance in breast cancer cells. A multifactorial approach was employed, with comparisons of cellular proliferation between 2D and 3D cell cultures, diffusion of doxorubicin into 3D cell cultures, adhesion-mediated signal transduction and combination therapy evaluation undertaken. We report the outcomes obtained using advanced cell culture conditions to elucidate microenvironment-based mechanisms that modulate doxorubicin resistance in representative breast cancer cell lines.

## Methods

### Cell culture conditions

The MCF-7 (HTB-22™) and MDA-MB-231 (HTB-26™) cell lines utilised in this study were obtained from the American Type Culture Collection. Cells were incubated at 37 °C in a humidified incubator inclusive of 5% carbon dioxide in phenol red-free DMEM/F12 with 10% heat inactivated fetal bovine serum (Life Technologies).

### 2D and 3D cell culture assays

The cell culture assays were performed as previously published [11, 14]. Briefly, for 3D cell cultures 1000 MDA-MB-231 cells or 5000 MCF-7 cells were seeded in 384-well microplates (CellCarrier; PerkinElmer) on top of 7.6 mg/ml Growth Factor Reduced (GFR) Matrigel™ or 7.5 mg/ml PuraMatrix™ (Becton Dickinson Biosciences). Once an average spheroid size of approximately 50-100 μm in diameter was reached (up to a 6-day incubation) drug was applied for a period of 6 days. For 2D monolayer assays, 600 cells per well were seeded into 384-well CellCarrier microplates. Following a 24-h incubation, cells were exposed to doxorubicin and incubated for 6 days. A dose-response curve for doxorubicin was performed with final concentrations per well between 0.0002 μM and 200 μM (3D: 12 point dose response curve, 2D: 20 point dose response curve). Doxorubicin (Tocris Bioscience) was stored at a concentration of 50 mM in dimethyl sulfoxide (DMSO) at −20 °C.

At the conclusion of both assays, a final concentration of 600 μM resazurin (Sigma-Aldrich) was added to each well and incubated for 4–6 h at 37 °C. The fluorescence intensity was detected using an EnVision™ multilabel plate reader (excitation 530 nm, emission 595 nm; PerkinElmer). In addition, 3D cell cultures were imaged using an Operetta™ High Content Imaging System (PerkinElmer) at the assay conclusion with and without the addition of 2 μM calcein AM dye (Life Technologies).

### Cellular proliferation

The cellular proliferation rates of breast cancer cell lines cultured in 2D and 3D were assessed via a resazurin reduction assay at 37 °C in a humidified incubator (5% carbon dioxide). A final concentration of 600 μM resazurin was added to each assay well at each specified time period throughout the assay duration, and fluorescence detected on an EnVision multilabel plate reader. Fluorescent intensity values were used to calculate cellular proliferation rates (based on a linear relationship between cell number and total fluorescent units).

### Doxorubicin diffusion

To study the diffusion of doxorubicin into 3D cell culture spheroids, a 0.5-2 μM range of concentrations of the inherently fluorescent doxorubicin was applied to cells in 3D cultures and imaged. The total exposure time for breast cancer 3D cell cultures to doxorubicin prior to live cell imaging was between 6 and 72 h. Nuclei were stained with Hoechst 33,342 and incubated for 2 h at 37 °C in a humidified incubator inclusive of 5% carbon dioxide. Using the 10× objective on an Opera™ High-Content Screening System (PerkinElmer), Z-slices at 10 μm intervals through the spheroids were captured. Analysis of a central Z-slice of a spheroid was undertaken using the radial profile plugin in ImageJ (http://rsbweb.nih.gov/ij/plugins/radial-profile.html). Values generated from ImageJ were graphed using Graphpad Prism.

### Automated confocal image acquisition and analysis

To assess pro-survival protein expression in cells cultured in 2D and 3D conditions, assays were prepared as described above. Doxorubicin (2D: 90 nM; 3D: 720 nM) or the vehicle control were applied to cells for 24, 48 and 72 h time points. For immunostaining, 2D and 3D cell cultures were fixed for 10–20 min using 4% paraformaldehyde (PFA; Sigma-Aldrich) followed by washing with phosphate buffered saline (PBS; Life Technologies). Cell cultures were blocked with 2% Bovine Serum Albumin (BSA; Sigma-Aldrich) and 0.5% Triton X-100 (Sigma-Aldrich) in PBS. Anti-Bcl-xL (Cell Signaling) and anti-Bcl-2 (Life Technologies) primary antibodies in blocking buffer were added to wells and incubated at room temperature for 16–20 h. Wells were rinsed with PBS, followed by the addition of Hoechst 33,342 (Life Technologies), Alexa Fluor® secondary antibody (Life Technologies) and Alexa Fluor® phalloidin (300 units; Life Technologies) or Cell Mask (Life Technologies) in blocking solution for 2 h at room temperature. Cells were then washed with PBS before imaging.

Confocal fluorescent images were acquired from each assay well using an Opera High Content Screening System. Image analysis was performed with the Columbus™ Image Analysis and Data Management Software (PerkinElmer). In 2D cell cultures, expression levels of the protein of interest were measured by calculating the average intensity (in a region of interest) of a single image plane through a cell monolayer at 20× lens magnification. In 3D cell cultures, expression levels of the protein of interest were measured by calculating the average intensity (in a region of interest) of either a single central slice or a maximum projection image constructed from 10 μm confocal image stack at 10× lens magnification. Representative high magnification images of 3D cultures were acquired at 20× lens magnification to visualise protein localisation. All quantification from image analysis protocols were performed on unmodified files.

### β1-integrin inhibition

The characterisation of β1-integrin expression and morphological properties of the spheroid following inhibition were determined. β1-integrin function blocking antibody (P5D2; R&D Systems) and the equivalent isotype antibody for use in control wells (R&D Systems) were mixed into GFR Matrigel at a concentration of 1.5 μg/ml and layered into a 384-well microplate. The P5D2 antibody has previously been demonstrated to inhibit the function of β1-integrin at a concentration of 1.5 μg/ml [15]. Breast cancer cells were seeded into wells as described above. Cells were exposed to doxorubicin (720 nM; final concentration) for 72 h once average spheroids size reached 50-100 μm in diameter. Immunostaining, confocal imaging and analysis were performed as described above utilising the anti-β1-integrin antibody (R&D Systems) for the β1-integrin expression experiments. Live cell differential interference contrast (DIC) imaging was performed on a CellR microscope (Olympus Life Sciences) with image analysis completed via ImageJ Version 1.46 h (area; images were flat field corrected and then analysed [14] and the AxioVision™ software (longest diameter; ‘length tool’).

Fifteen microlitres of 7.6 mg/ml GFR Matrigel with 1.5 μg/ml of β1-integrin function blocking antibody or the equivalent isotype antibody for use in control wells (R&D Systems) were added to wells of a 384-well microplate. One-thousand MDA-MB-231 cells were added to each well and incubated at 37 °C in a humidified incubator inclusive of 5% carbon dioxide for 6 days (media changes after 3 days incubation). Doxorubicin was applied (0.07-5 μM) for a period of 72 h. At the assay conclusion, a final concentration of 600 μM resazurin was added to each well and incubated for 6–14 h at 37 °C in a humidified incubator inclusive of 5% carbon dioxide. The fluorescence intensity was measured by an EnVision multilabel plate reader. Representative DIC images were acquired on a CellR microscope.

### Statistical analyses

The statistical analyses for this study were completed in Graphpad Prism using an unpaired t-test or a one-way ANOVA, followed by a Bonferroni or a Tukey *post-hoc* test.

## Results

### Doxorubicin activity in 2D vs. 3D cell culture conditions

A study was undertaken to evaluate doxorubicin resistance mechanisms exhibited by cells in a 3D ECM-based breast cancer model. Initially, experimentation was undertaken to ascertain if, and to what extent, culturing cells in 3D conditions impacted on doxorubicin activity. The potency (half maximal inhibitory concentration; IC_50_ value), together with combined efficacy and potency (area under the curve; AUC) were measured.

**Fig. 1:**
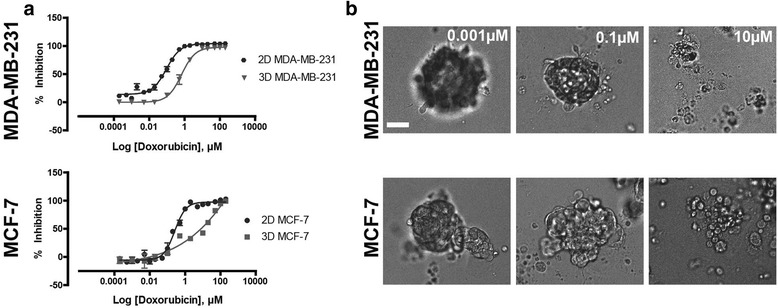
The anti-cancer activity of doxorubicin on MDA-MB-231 and MCF-7 breast cancer cell lines. (**a**) Dose-response curves of 2D and 3D MDA-MB-231 and MCF-7 cultured cells. (**b**) Brightfield morphology of 3D cultured breast cancer cells following exposure to doxorubicin. Scale bar = 50 μm. Data represent mean ± standard deviation

Doxorubicin was significantly (*p* ≤ 0.001) more potent against the breast cancer cells grown in 2D cultures in comparison to those cultured in a 3D ECM-based model (Table 1). Furthermore, both MCF-7 and MDA-MB-231 cells exhibited significantly reduced (*p* ≤ 0.0001) efficacy upon doxorubicin application in 3D conditions in comparison to 2D culture (Table 1). Not only were there significant differences in the potency and efficacy of doxorubicin evaluated against breast cancer cell lines in 2D and 3D culture conditions, the shape of the MCF-7 dose-response curve demonstrated variances in the cellular response to drug in 3D cell culture compared to 2D cell culture (Fig. 1a). The morphological response to doxorubicin observed for the breast cancer cells in the 3D culture system indicated a substantial deterioration of the 3D cellular architecture at 10 μM (Fig. 1b). The data indicates that selected breast cancer cell lines cultured in 3D conditions are more resistant to doxorubicin in comparison to those cells cultured as 2D monolayers.

**Table 1:** The half-maximal inhibition (IC_50_) and area under the curve (AUC) values for MDA-MB-231 and MCF-7 cells cultured in 2D and 3D cell culture

Doxorubicin	MDA-MB-231		MCF-7	
	2D	3D	2D	3D
Drug IC50 (nM)	87.7 ± 10.6	636.0 ± 160.3***	225.2 ± 64.2	10,000^#^****
AUC (units)	370.4 ± 17.1	244.7 ± 13.7****	291.4 ± 7.8	174.4 ± 9.1****

Significance values are: *p* ≤ 0.001 (***), *p* ≤ 0.0001 (****).^#^GraphPad Prism unable to calculate IC_50_ value, estimated from raw data. Data represent mean ± standard deviation, *n* = 3

### Cellular proliferation in 2D vs. 3D cell culture conditions

**Fig. 2:**
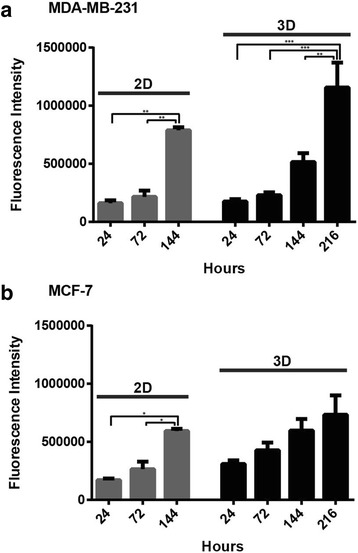
Cellular proliferation of 2D and 3D cell cultures over time. Total well proliferation of MDA-MB-231 (**a**) and MCF-7 (**b**) cells in 2D and 3D cultures. Significance values are: *p* ≤ 0.05 (*), *p* ≤ 0.01, (**), *p* ≤ 0.001 (***). Data represent mean ± standard error

Investigation into the doxorubicin resistance observed in MCF-7 and MDA-MB-231 cell lines cultured in 3D was undertaken, with initial research conducted on the rates of cellular proliferation between cells cultured in traditional 2D monolayer and 3D cell cultures. Utilising a metabolic indicator dye, previously demonstrated to reflect cell number [14, 16], the number of cells per well under both culture conditions were measured at specific intervals (24 to 72 h) over 6 day (2D) and 9 day (3D) time frames. Outcomes demonstrated that cellular propagation occurred in both the 2D and 3D cell culture systems for both MCF-7 and MDA-MB-231 cell lines (Fig. 2a, b). The total well fluorescence intensity indicated a reduction in the doubling time for MDA-MB-231 (2D: 47.6 ± 10.2, 3D: 69.5 ± 7.2) and MCF-7 (2D: 55.2 ± 3.3, 3D: 190.9 ± 33.9; *p* ≤ 0.05) cells grown in 3D cell culture compared to those cultured on plastic substrata. Overall, there was a temporal increase in cell number for both breast tumour cell lines in both 2D and 3D culture conditions, and cellular proliferation was decreased in 3D cell cultures for both breast cancer cell lines tested.

### Doxorubicin diffusion in 3D cell cultures

**Fig. 3:**
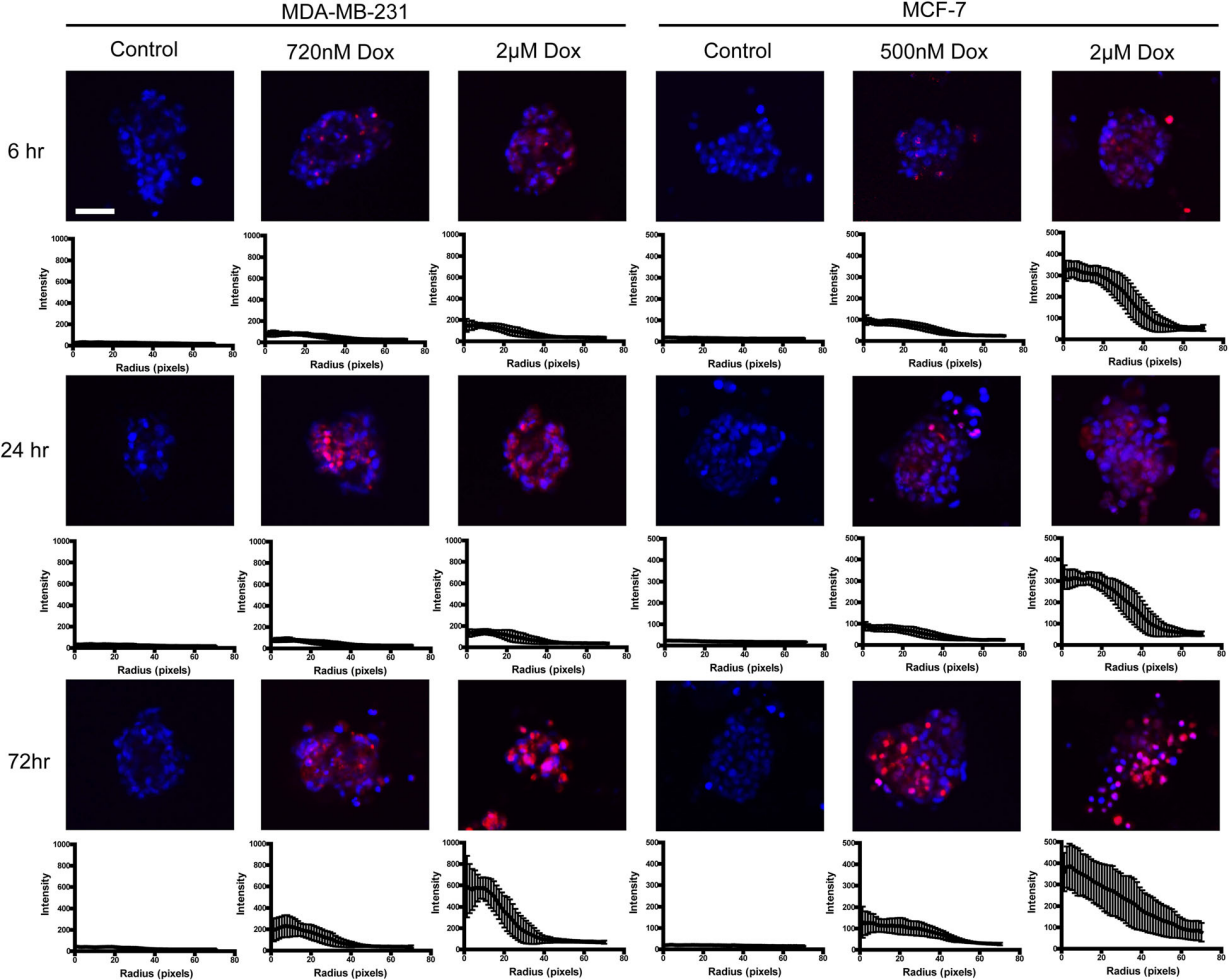
Diffusion of doxorubicin within 3D cell cultures for MDA-MB-231 and MCF-7 cell lines. The vehicle (negative control) and doxorubicin concentrations were evaluated. Images captured (central slice shown) at 6, 24 and 72 h time points. Image analysis was completed utilising the radial profile plugin in ImageJ. Scale bar = 50 μm

The diffusion of doxorubicin within 3D cell cultures was examined to determine if limited cellular exposure was a contributing factor to the doxorubicin resistance observed in breast cancer cells. The diffusion of doxorubicin within 3D cultures of both the MCF-7 and MDA-MB-231 cell lines was investigated at 6, 24 and 72 h time points. Results demonstrate that doxorubicin was detected within both MCF-7 and MDA-MB-231 spheroids at the 6 h time point and the levels of doxorubicin were observed to increase within the breast cancer spheroids over time, particularly upon exposure to 2 μM doxorubicin (Fig. 3).

### Impact of ECM molecules on doxorubicin resistance

**Fig. 4:**
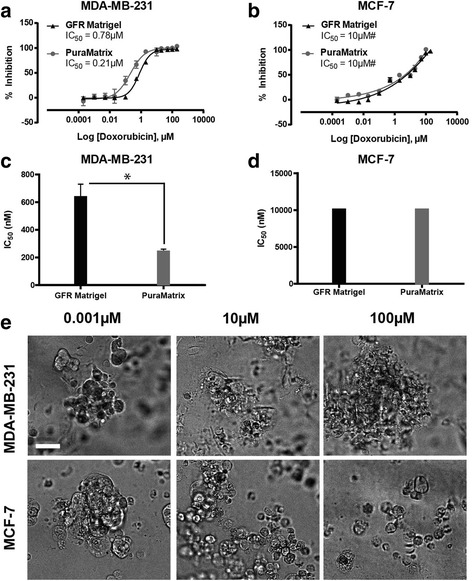
Breast cancer cell line response to doxorubicin exposure when cultured in the absence of ECM proteins. Doxorubicin dose-response activity on MDA-MB-231 (**a**) and MCF-7 (**b**) cells cultured on PuraMatrix™ or Growth Factor Reduced (GRF) Matrigel™. Collective IC_50_ results of doxorubicin activity on MDA-MB-231 (**c**) and MCF-7 (**d**) cells cultured in the presence of PuraMatrix or GFR Matrigel. (**e**) Morphology of breast cancer cells cultured on PuraMatrix and exposed to doxorubicin (0.001-100 μM). # GraphPad Prism unable to calculate IC_50_ value, estimated from raw data. Significance values are: *p* ≤ 0.05 (*). Scale bar = 50 μm. Data are mean ± standard deviation

To investigate the impact cell attachment to the ECM had on cells grown in 3D culture with GFR Matrigel, a synthetic hydrogel, PuraMatrix, was employed to promote spheroid formation in the absence of specific ECM proteins (e.g. laminin, collagen IV). Doxorubicin was applied to spheroids cultured on GFR Matrigel and PuraMatrix and comparisons of the cellular response conducted. The potency of doxorubicin was significantly increased (*p* ≤ 0.05) against the MDA-MB-231 cell line cultured in 3D on PuraMatrix when compared to GFR Matrigel (Fig. 4a, c). However, there were no significant differences (*p* > 0.05) in drug sensitivity detected when the MCF-7 cells were cultured on the alternative matrix (Fig. 4b, d). The morphology of cells grown on PuraMatrix (Fig. 4e) was similar to those cultured on GFR Matrigel (Fig. 1b). Thus, the attachment of cells to selected ECM proteins may play a role in mediating drug resistance in MDA-MB-231 cells.

### Modulation of pro-survival proteins in MDA-MB-231 cells cultured in 3D ECM-based conditions

**Fig. 5:**
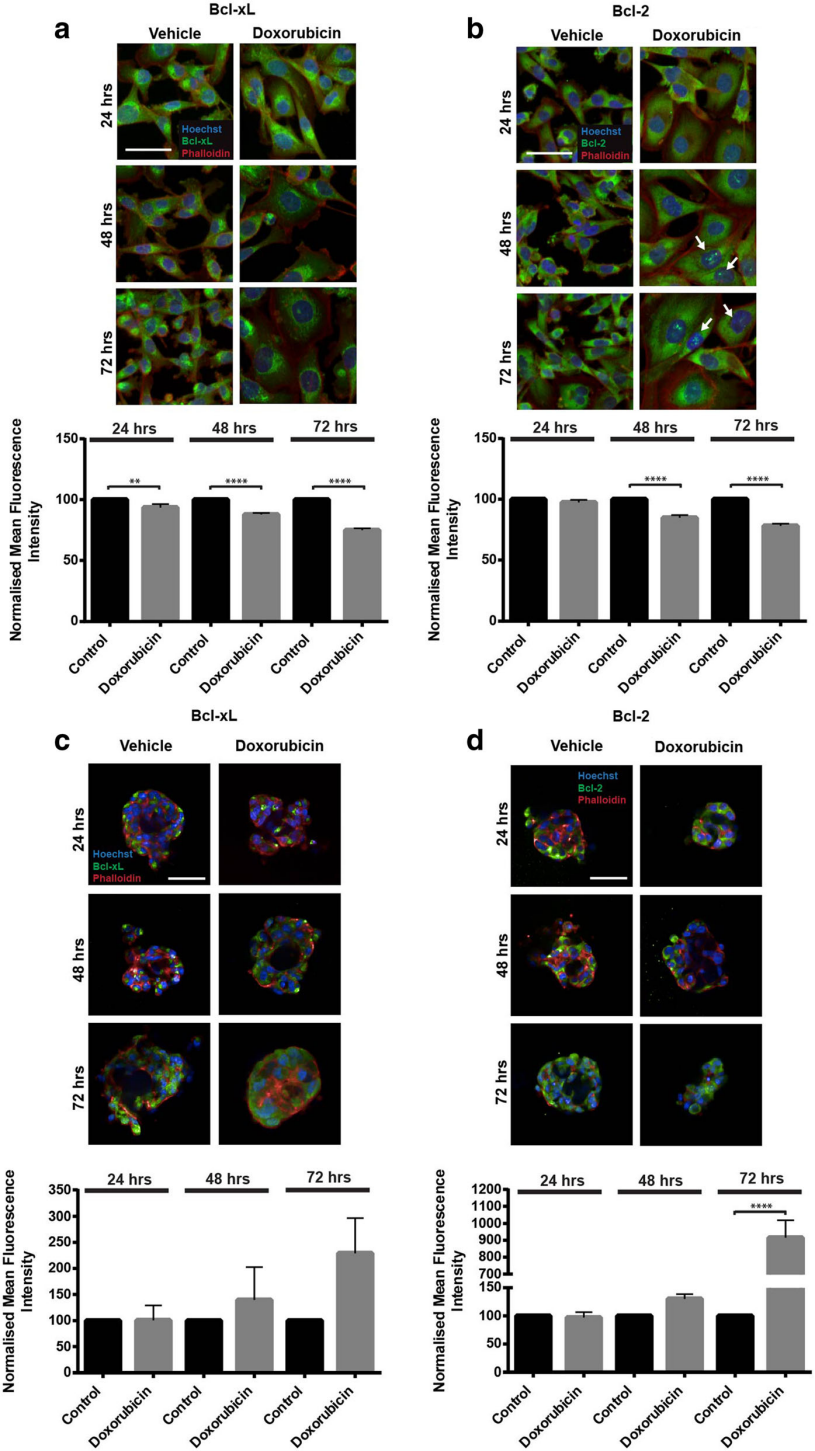
Modulation of pro-survival proteins in 2D and 3D cell culture. Bcl-xL (**a**) and Bcl-2 (**b**) protein expression in MDA-MB-231 monolayer cultured cells over time following exposure to doxorubicin. Bcl-xL (**c**) and Bcl-2 (**d**) protein expression in MDA-MB-231 cells in 3D culture conditions over time following exposure to doxorubicin. Arrows indicate nuclear localisation of Bcl-2. Significance values are: *p* ≤ 0.01 (**), *p* ≤ 0.0001 (****). Scale bar = 50 μm. Data represent mean ± standard error

To investigate a potential survival advantage of cells cultured in 3D conditions in an ECM-rich microenvironment, two proteins integral in mediating cell survival, Bcl-2 and Bcl-xL, were examined. Results show that Bcl-2 and Bcl-xL were expressed in untreated and treated MDA-MB-231 cells at 24, 48 and 72 h time points. Following exposure to doxorubicin, there was a significant reduction (*p* ≤ 0.0001) in the levels of both Bcl-xL and Bcl-2 (48 and 72 h following application) with time in MDA-MB-231 cells cultured in 2D conditions (Fig. 5a, b). In addition, it was observed that Bcl-2 expression was localised to the nucleus of doxorubicin-treated cells following 48 and 72 h of exposure. The presence of Bcl-2 in the nucleus has been observed previously, and associated with a pro-apoptotic function [17, 18]. Conversely, the expression levels of the pro-survival proteins in doxorubicin-treated cells in 3D cultures were equivalent or greater than untreated cells, particularly at the 72 h time point (Bcl-2, *p* ≤ 0.0001; Fig. 5c, d). Overall, the cellular levels of pro-survival proteins observed in the 2D model were the opposite trend to the levels measured in the 3D ECM-based model.

### Impact of β1-integrin signalling inhibition on doxorubicin sensitivity

**Fig. 6:**
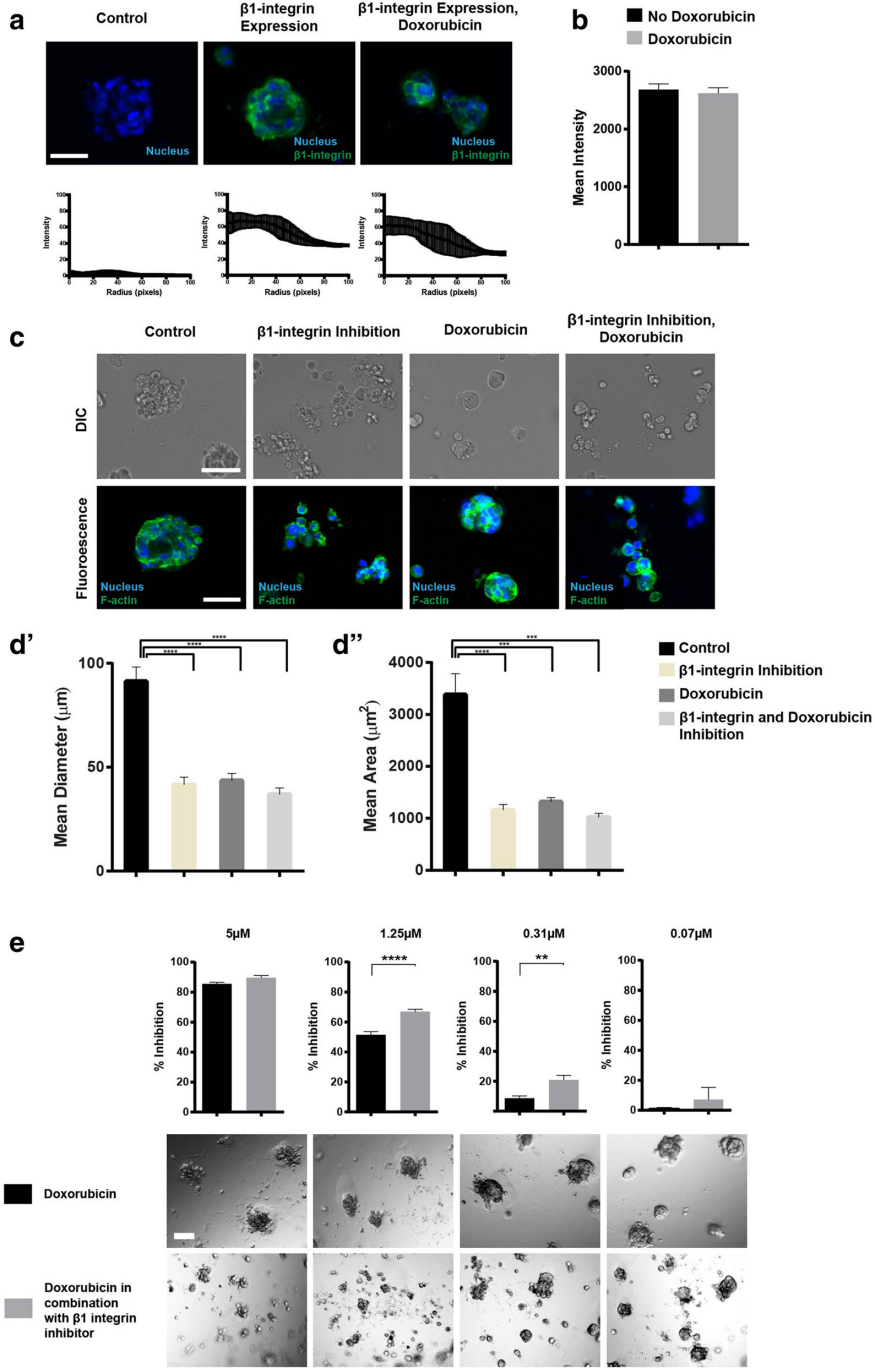
Response of MDA-MB-231 cells cultured in three-dimensions to doxorubicin in combination with β1-integrin inhibition. (**a**) Expression of β1-integrin in MDA-MB-231 cells grown in 3D conditions; green fluorescence represents β1-integrin and blue fluorescence (Hoechst) represents nuclear staining. Intensity of β1-integrin staining is located below the corresponding image. Analysis was completed using the radial profile plugin in ImageJ. (**b**) The total mean intensity of β1-integrin staining in 3D cell cultures between untreated and treated (720 nM doxorubicin) cultures. (**c**) Morphological response of 3D cultures consisting of MDA-MB-231 cells to inhibitory agents; green fluorescence corresponds f-actin (phalloidin) and blue fluorescence (Hoechst) represents nuclear staining. Size of MDA-MB-231 3D cultures: mean diameter (**d’**) and mean area (**d”**), following exposure to inhibitory agents. (**e**) β1-integrin signalling was inhibited and combined with various concentrations of doxorubicin: 5 μM, 1.25 μM, 0.31 μM, 0.07 μM. Significance values are: *p* ≤ 0.01 (**), *p* ≤ 0.001 (***), *p* ≤ 0.0001 (****). Scale bar = 100 μm. Data represent mean ± standard error

The involvement β1-integrin signalling has on mediating the resistance of MDA-MB-231 cells grown in 3D cell culture in the presence of ECM proteins was investigated. As a result, the potential role of integrin signalling in doxorubicin resistance on MDA-MB-231 cells was evaluated. MDA-MB-231 cells express β1-integrin, the binding partner for various α-chain integrin heterodimers, in approximately equal quantities with and without the presence of doxorubicin (Fig. 6a, b). When β1-integrin to ECM protein binding was inhibited by inclusion of a function blocking antibody into the GRF Matrigel, morphological modifications were observed in 3D MDA-MB-231 cell cultures, including lack of spheroid integrity (Fig. 6c). The size of these treated 3D cell culture aggregates were measured and results show that inhibition with the β1-integrin function blocking antibody and/or doxorubicin resulted in significant *(p* ≤ 0.001) reductions in size (diameter and area) compared to the untreated control (Fig. 6d’, d”). To determine the therapeutic potential of blocking β1-integrin signalling in the presence of doxorubicin, the function of β1-integrin on MDA-MB-231 cells was blocked in combination with doxorubicin. Results show there was a dose-dependent enhanced (*p* ≤ 0.01) sensitivity of MDA-MB-231 cells in 3D culture to doxorubicin (Fig. 6e). These results suggest that blocking the function of β1-integrin prior to the addition of doxorubicin enhanced the efficacy of doxorubicin in a dose-dependent manner.

## Discussion

Doxorubicin remains one of the most active and widely used chemotherapy agents in the treatment of early and advanced breast cancer. However, tumour resistance has limited the effectiveness of the agent in single drug treatment regimes. The exact mechanisms behind the resistance are still poorly understood with in vitro studies using breast cancer cell models often lacking clinical relevance. The data acquired during this research demonstrate that ECM-to-cell specific elements can significantly affect breast cancer cell resistance to doxorubicin. Through the evaluation of these drug resistance mechanisms, cellular processes have been identified that may be relevant to in vivo anti-breast cancer doxorubicin activity.

The mechanisms of doxorubicin resistance observed in the 3D cell culture experiments conducted in our study were explored further. The reduced growth rate of breast cancer cells in spheroid cultures potentially plays a role in altering the inhibitory activity of doxorubicin, as the cytotoxicity may be less effective in cells with a slower doubling time when compared to cells that proliferate more quickly. Previous studies have also noted reduced proliferation rates in cancer cells grown in 3D ECM-based conditions compared to 2D cell culture [19, 20]. Spheroids cultured in the present study may not be large enough to have distinct cellular proliferation gradients throughout the spheroids (quiescent in the spheroid centre and active doubling at the spheroid periphery), such as when spheroids above the size of 200 μm in diameter are generated [21]. However, altered proliferation rates still may occur in spheroids sized below 150 μm [22]. Therefore, a reduced proliferation rate of cells grown under 3D culture conditions may be a contributing factor to the doxorubicin resistance observed in a 3D ECM-based system compared to the reciprocal 2D study.

We also examined the diffusion of doxorubicin through the cell layers within a spheroid. These studies illustrated convincingly that the lack of doxorubicin exposure to cells within 3D structures is not likely to be the cause of the observed drug resistance. The conditions in vivo are more complex, in particular, the delivery of drug to tissue via vasculature followed by the subsequent diffusion to tumour cells, however studying drug diffusion with in vitro 3D models permits evaluation of drug distribution [23, 24]. Thus, the use of the 3D model system as a tool allowed the evaluation of anti-cancer agent diffusion, a factor demonstrated as unlikely to be contributing to doxorubicin resistance in the breast cancer cells examined.

De novo resistance to doxorubicin in MDA-MB-231 and MCF-7 cells was investigated by culturing the cells in the absence of exogenous ECM. It was observed that resistance to doxorubicin was cell line-dependent, indicating that multiple mechanisms may influence breast cancer cell behaviour in complex microenvironments. In the absence of the ECM proteins present in PuraMatrix, MDA-MB-231 cells were significantly more sensitive to doxorubicin than when compared to the same cell line cultured with GRF Matrigel. The interactions between the tumour and its microenvironment can affect cellular signalling, which has been reported to impact on the response of tumour cells to therapy [25, 26]. Specifically, ECM-to-tumour cell mediated resistance to chemotherapeutics has been shown in a range of different cancers [27–29]. Integrins play a central role in ECM-to-cell attachment and are involved in instigating downstream signal transduction in cells resulting in modulation of several cellular processes, including apoptosis [30]. Thus, cellular survival proteins downstream of integrin signal transduction were examined.

Pro-survival proteins have been identified as factors involved in the cellular resistance of anti-cancer agents against additional cancer types. For instance, T cell acute lymphoblastic leukaemia cell lines displayed resistance to doxorubicin, which was demonstrated to be dependent on Mcl-1 pro-survival protein expression through α2β1 integrin signalling [31]. Furthermore, Muranen et al. [32] showed that cellular attachment to the ECM permitted cell survival of ovarian cells cultured in 3D conditions upon exposure to BEZ235, an Akt/mammalian target of rapamycin (mTOR) inhibitor. Increased expression of Bcl-2 and Bcl-xL were detected in the cells situated at the periphery of the spheroid, specifically those in contact with the ECM. Thus, cell-to-ECM contacts can modulate anti-cancer drug activity, potentially through the mediation of pro-survival protein levels.

In addition to demonstrating the influence of cell-to-ECM interactions in the cell culture microenvironment, and an altered trend in pro-survival protein regulation, we showed that a combination therapy approach was an effective means of targeting MDA-MB-231 breast cancer cells. One of the therapeutic targets was β1-integrin. The β1-integrin subunit interacts with a range of α subunits enabling 12 combinations of heterodimers that attach to ECM proteins [33]. MDA-MB-231 cells express a variety of integrin subunits including α2, α3, α5, α6 and β1 [34]. Doxorubicin was more effective against MDA-MB-231 cells when ECM-cell signalling was disrupted. Our data complements pre-clinical evidence that inhibiting β1-integrin is a therapeutic strategy for targeting breast cancer tumours [35] and enhances anti-tumour activity following radiation therapy [36].

## Conclusions

In summary, breast cancer cells cultured in 3D demonstrated significant resistance against doxorubicin in comparison to 2D cell cultures. Understanding how chemoresistance arises in breast cancer cells in more biologically relevant models (that aim to better reflect resistance patterns observed in tumours in vivo) may provide improved direction for drug discovery programs. To determine the potential mechanisms of resistance, several elements influencing doxorubicin sensitivity were evaluated. A decrease in cellular proliferation in 3D cell cultures and cell-to-ECM adhesion, perhaps through the up-regulation of pro-survival proteins, were implicated in mediating doxorubicin resistance. Inhibition of β1-integrin or associated signalling proteins may prove therapeutically beneficial in combination with doxorubicin.

## Abbreviations

2D: Two-dimensional
3D: Three-dimensional
ECM: Extracellular matrix
mTOR: Mammalian target of rapamycin
